# Influenza in Refugees on the Thailand–Myanmar Border, May–October 2009

**DOI:** 10.3201/eid1609.100220

**Published:** 2010-09

**Authors:** Paul Turner, Claudia L. Turner, Wanitda Watthanaworawit, Verena I. Carrara, Bryan K. Kapella, John Painter, François H. Nosten

**Affiliations:** Author affiliations: Shoklo Malaria Research Unit, Mae Sot, Thailand (P. Turner, C.L. Turner, W. Watthanaworawit, V.I. Carrara, F.H. Nosten);; Mahidol–Oxford Tropical Medicine Research Unit, Bangkok, Thailand (P. Turner, C.L. Turner, W. Watthanaworawit, V.I. Carrara, F.H. Nosten);; University of Oxford, Oxford, UK (P. Turner, C.L. Turner, F.H. Nosten);; Centers for Disease Control and Prevention, Atlanta, Georgia, USA (B.K. Kapella, J. Painter)

**Keywords:** Influenza, human, disease outbreaks, pneumonia, viruses, bacteria, pandemic (H1N1) 2009, refugees, Thailand, research, *Suggested citation for this article*: Turner P, Turner CL, Watthanaworawit, Carrara VI, Kapella BK, Painter J, et al. Influenza in refugees on the Thailand–Myanmar border, May–October 2009. Emerg Infect Dis [serial on the Internet]. 2010 Sep [*date cited*]. http://dx.doi.org/10.3201/eid1609.100220

## Abstract

We describe the epidemiology of influenza virus infections in refugees in a camp in rural Southeast Asia during May–October 2009, the first 6 months after identification of pandemic (H1N1) 2009 in Thailand. Influenza A viruses were detected in 20% of patients who had influenza-like illness and in 23% of those who had clinical pneumonia. Seasonal influenza A (H1N1) was the predominant virus circulating during weeks 26–33 (June 25–August 29) and was subsequently replaced by the pandemic strain. A review of passive surveillance for acute respiratory infection did not show an increase in acute respiratory tract infection incidence associated with the arrival of pandemic (H1N1) 2009 in the camp.

## Abstract 2

TOC Summary: Influenza viruses can be identified in up to 22% of patients who have acute respiratory infections.

Pandemic (H1N1) 2009 emerged in April 2009 and subsequently spread around the globe. The World Health Organization issued a pandemic declaration on June 11, 2009 (*1**,**2*). By October 25, 2009, >440,000 laboratory-confirmed cases, including >5,700 deaths, had been reported to WHO (*3*). The first case of pandemic (H1N1) 2009 infection was diagnosed in Thailand on April 28, 2009, and subsequently the virus was detected in all provinces. The Thailand Ministry of Public Health reported 27,639 confirmed cases and 170 deaths as of October 10, 2009 (*4*). Myanmar (Burma) reported its first confirmed case of pandemic (H1N1) 2009 infection during the week beginning July 5, 2009, and by the end of October 2009 had reported <100 confirmed cases with no deaths (*5*). Although most infections caused by this new virus have been mild, severe disease has been reported, particularly in young adults (*6*).

Data regarding the effect of influenza in rural areas of the developing world are scarce, as are etiologic data from refugee populations (*7**–**9*). A recent review of published reports from Southeast Asia concluded that influenza infection may be identified in up to 26% of outpatients with febrile illness and in 14% of hospitalized patients with pneumonia (*10*). In Thailand, seasonal influenza virus activity peaks during the rainy season (June– September), with smaller peaks occurring during the cold months (January and February) (*11*). Incidence of influenza infections in Thailand was 64–91 cases/100,000 persons per year during 1999–2002; the influenza-related hospitalization rate was 21/100,000 persons during 1999 (*11*). Influenza infections in Myanmar are also seasonal; cases are documented predominantly in the rainy season (May–October) (*12**–**14*). Incidence data for influenza virus infections in Myanmar are not readily available.

Of 15.2 million refugees worldwide, approximately one third live in camps (*15*). These refugees often live in crowded conditions and have contact with populations from the host country and the country of origin, where public health infrastructure and surveillance may be poor (*16**,**17*).

Approximately 150,000 refugees from Myanmar are housed in several camps on the Thailand–Myanmar border. Maela Temporary Shelter (Maela, Thailand) is the largest of these camps, with a population of >40,000, predominantly of the Karen ethnic group, housed in a 4-km^2^ area (*18*). This camp is located in the hills adjoining the Myanmar border, ≈500 km northwest of Bangkok, and has been in operation since 1984. Primary health and sanitation services are provided by nongovernmental organizations. A field hospital with an inpatient area and 2 outpatient clinics provide free healthcare to the camp’s population, who do not have access to healthcare facilities outside of the camp. Acute respiratory infection (ARI) is a common cause of illness in Maela, but the proportion of infections caused by influenza viruses is unknown. Seasonal influenza vaccinations and antiviral medicines are not readily available in the camp or the surrounding community.

In 2007, the US Centers for Disease Control and Prevention (CDC) and Shoklo Malaria Research Unit (www.shoklo-unit.com) established a laboratory-enhanced ARI surveillance system in Maela. Pilot data were obtained during late 2007, and formal surveillance began in 2008 with a 2-day-per-week patient review in the outpatient department of Aide Medicale Internationale Hospital. In 2009, daily patient reviews were carried out in outpatient (from January 2009) and inpatient (from April 2009) departments. We report the results of this surveillance during May–October 2009 and describe the impact of the current influenza pandemic in this rural refugee population. Data from our surveillance activities in 2008, as well as passively collected ARI incidence data, are included for comparison.

## Methods

From May 1 through October 31, 2009, trained local field workers visited the hospital in Maela daily (Monday–Saturday). Patients whose illnesses met clinical case definitions for influenza-like illness (ILI) or pneumonia (Table 1) were identified by clinic staff at the time of examination, and these patients were asked to complete an additional clinical interview. Inpatient and outpatient department cases were included in the surveillance. From July 27 through October 31, 2009, original clinical case definitions were modified to capture each patient who had a history of fever during the current illness but who was not febrile at the clinic visit (either because of the intermittent nature of fever or self-administration of antipyretics).

**Table 1:** Clinical case definitions for influenza virus infections in Maela Temporary Shelter, Thailand, May–October 2009*

Condition (age, y)	Strict case definition (up to 2009 Jul 27)	Expanded case definition (from 2009 Jul 28)
Influenza-like illness	Fever >38°C AND cough or sore throat AND does not meet criteria for pneumonia	Fever >38°C (or history of fever) AND cough or sore throat AND does not meet criteria for pneumonia
Pneumonia (<5)	Pneumonia: cough or difficulty breathing AND increased respiratory rate (as defined by the WHO IMCI [*19*])	No change
	Severe pneumonia: cough or difficulty breathing AND >1 of: lower chest wall in-drawing, nasal flaring, grunting	
Pneumonia (>5)	Fever >38°C AND cough or difficulty breathing AND abnormal chest examination	Fever >38°C (or history of fever) AND cough or difficulty breathing AND abnormal chest examination

*WHO, World Health Organization; IMCI, Integrated Management of Childhood Illness.

A nasopharyngeal aspirate (NPA) was collected from each patient; a sterile 8-French infant feeding tube was inserted into the nasopharynx and then withdrawn while suction was applied with a 20-mL syringe attached to the feeding tube. The nasopharyngeal secretions and the tip of the feeding tube were transferred to a 1-mL tube of viral transport medium and stored in a cool box until transfer, within 24 h, to a –80°C freezer before analysis.

All NPA specimens were subjected to a panel of real-time reverse transcription–PCR (rRT-PCR) assays for the following viruses: influenza A (separate primer/probe sets for influenza A [universal], pandemic [H1N1] 2009, seasonal subtype H1N1, and seasonal subtype H3N1 detection) (*20*); influenza B (CDC in-house assay [details available on request]); respiratory syncytial virus (RSV; CDC in-house assay [details available on request]); and human metapneumovirus (HMPV) (*21*). An internal control PCR specific for the human RNAseP gene was used to monitor sample adequacy and to detect the presence of PCR inhibitors (*22*). Positive and negative controls were included in each PCR run. A Rotorgene 6000 real-time PCR thermocycler (Corbett Life Science, Mortlake, New South Wales, Australia) and SuperScript III One-Step RT-PCR Kits (Invitrogen, Carlsbad, CA, USA) were used throughout. All laboratory work was conducted at the Shoklo Malaria Research Unit microbiology laboratory in Mae Sot, Tak Province, Thailand.

To compare virologic results from 2009 with our surveillance data from 2008, we subsequently restricted the 2009 dataset to match data collected in 2008 (i.e., we included only patients whose illnesses met the strict case definitions and who were sampled on either Monday or Tuesday in the outpatient department). Clinical and laboratory data collected in 2008 were identical to data collected in 2009.

To estimate the incidence of influenza-associated illness, we reviewed passive disease surveillance data collected by the hospital in Maela and collated by the Committee for Coordination of Services for Displaced Persons in Thailand. This surveillance system captured data only on patients visiting the hospital for treatment. The number and incidence rate (calculated by using monthly camp population census data) of clinically diagnosed upper respiratory tract infections (URTIs) and lower respiratory tract infections (LRTIs) were reported by month. No information was available to determine the number of ILI cases; therefore, we could not estimate the proportion of URTIs caused by influenza viruses in Maela. However, because most LRTIs reported are likely to be clinical pneumonia, we estimated the incidence of influenza-associated pneumonia as the incidence of LRTI multiplied by the percentage of pneumonia patients with specimens positive for influenza A. To determine the effect of pandemic (H1N1) 2009 on overall case numbers, we compared 2008 data with 2009 data.

### Ethics

The Human Studies Oversight and Review Team of CDC reviewed the surveillance project and declared it to be a nonresearch activity, as defined by US 45 CFR 46.102(d). Therefore our study was exempt from the need for full review by an institutional review board.

### Statistical Analysis

All statistical analyses were performed by using STATA version 10.1 software (StataCorp, College Station, TX, USA). Categorical variables were analyzed by using the Fisher exact test; continuous variables were analyzed by using the Wilcoxon rank-sum test (because none were normally distributed). Two-tailed p values <0.05 were considered significant. Epidemiologic week numbers were calculated by using standard criteria (*23*).

## Results

**Figure 1:**
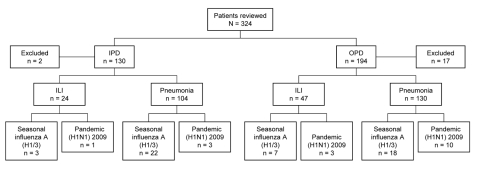
Influenza surveillance summary for Maela Temporary Shelter, Thailand, May–October 2009. IPD, inpatient department; OPD, outpatient department; ILI, influenza-like illness.

During May 1–October 31, 2009, a total of 324 patients were included in the surveillance. Of these, 19 were excluded from further analysis; 18 patients did not meet the clinical case definitions, and no NPA specimen was received for 1 patient (Figure 1).

Pneumonia was diagnosed for 234 (77%) of the 305 eligible patients, and ILI was diagnosed for 71 (24%). For patients with pneumonia, median age was 2.0 years (range 0.1–68 years) and 55% were male; for those with ILI, median age was 1.4 years (range 0.2–10 years) and 54% were male.

**Figure 2:**
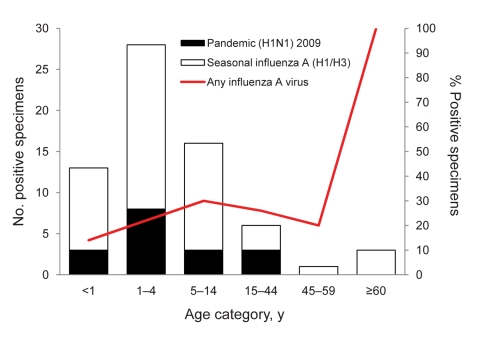
Age distribution of patients from whom specimens were positive for seasonal influenza (n = 50) or pandemic (H1N1) 2009 (n = 17) in Maela Temporary Shelter, Thailand, May–October 2009.

Fifty seasonal influenza A infections and 17 pandemic (H1N1) 2009 infections were detected by rRT-PCR. Forty-nine of the 50 seasonal influenza A infections were subtyped as H1N1; one was subtype H3N1 (Figure 2; Table 2). No influenza B infections were detected. Median age of patients with seasonal influenza A and pandemic (H1N1) 2009 was 3 years for both groups, but pandemic (H1N1) 2009 was more restricted in age range (upper limit 27 years, compared with 68 years for seasonal influenza A). Influenza A virus was detected in 23% of patients who had pneumonia (seasonal influenza A, 17%; pandemic [H1N1] 2009, 6%) and in 20% of ILI cases (seasonal influenza A, 14%; pandemic [H1N1] 2009, 6%).

**Table 2:** Characteristics of patients with influenza A infection in Maela Temporary Shelter, Thailand, May–October 2009*

Characteristic	Seasonal influenza A (H1N1/H3N1)	Pandemic (H1N1) 2009
No. cases	50	17
Median age, y (range)	3.1 (0.3–68.0)	3.7 (0.4–27.0)
Sex ratio, M:F, no. (%)	31:19 (62:38)	10:7 (59:41)
Admitted to IPD, no. (%)	25 (50)	4 (24)
Diagnosis		
Influenza-like illness, no. (%)	10 (20)	4 (24)
Pneumonia, no. (%)	40 (80)	12 (76)

*IPD, inpatient department, Aide Medicale Internationale Hospital.

**Figure 3:**
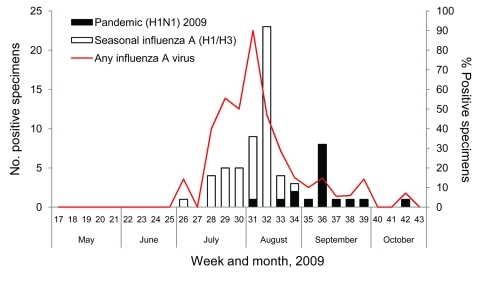
Influenza virus PCR results by week for Maela Temporary Shelter, Thailand, May–October 2009.

Seasonal influenza A activity spanned weeks 26–34 (June 28–August 29) and peaked in week 31 (August 2–8; the virus was detected in 80% of all NPA samples obtained from patients that week) (Figure 3). Pandemic (H1N1) 2009 was detected later, beginning on week 31 (August 3). Activity subsequently remained steady, with a maximum of 8 cases detected in a single week (15% of all patients sampled in week 36).

Seven dual virus infections were detected, all in children <14 years of age: 2 seasonal influenza A plus HMPV, 2 pandemic (H1N1) 2009 plus HMPV, and 3 pandemic (H1N1) 2009 plus RSV. Dual infections were observed significantly more frequently with pandemic (H1N1) 2009 than with seasonal influenza A (5/17 vs. 2/50; p = 0.003). Among patients admitted to the inpatient department, those with dual virus infection were not significantly more ill than those with influenza A infection alone (3/7 vs. 26/60; p = 1.0).

Illnesses for 205 (67%) patients met the strict case definition for ILI or pneumonia; 100 (33%) met only the expanded case definitions. Age distribution and proportion of influenza A viruses did not differ significantly between the strict and expanded case definition groups. However, a significantly higher proportion of patients with ILI (18/25 vs. 6/46; p<0.001) or pneumonia (99/180 vs. 5/54; p<0.001) whose illnesses met the strict case definition were hospitalized, which suggests that the expanded case definitions captured patients with milder illnesses.

Overall, at least 1 virus was detected in 175 (57%) patients (37/71 ILI, 138/234 pneumonia). HMPV and RSV accounted for 120/187 (54%) viruses detected. These viruses were detected in ILI cases (HMPV 23%,; RSV 17%) and pneumonia (HMPV, 21%; RSV 18%). RSV was detected significantly more often in children <5 years of age (48/221 vs. 6/84; p = 0.003) and was more age restricted than all other viruses.

In 2008, NPA samples were obtained from 74 patients meeting the case definitions on Mondays or Tuesdays in outpatient departments during May 1–October 31 (2 ILI, 72 pneumonia). An influenza virus was identified in 6 patients (3 influenza A, 3 influenza B); pneumonia was diagnosed for all. In 2009, samples were obtained from 35 patients with illnesses that met the strict case definitions in operation in 2008; patients were examined in outpatient departments on the same days of the week (3 ILI, 32 pneumonia). An influenza virus was detected in 9 patients (4 seasonal influenza A, 5 pandemic [H1N1] 2009); pneumonia was diagnosed in all.

**Figure 4:**
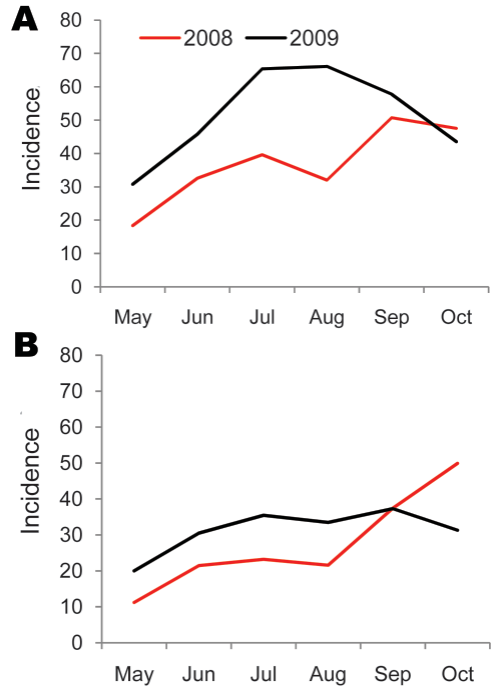
Incidence (per 1,000 population) of (A) upper respiratory tract infections (URTI) and (B) lower respiratory infections (LRTI) for Maela Temporary Shelter, Thailand, May–October 2008 and 2009. Passive surveillance data from Committee for the Coordination of Services for Displaced Persons in Thailand.

Committee for Coordination of Services for Displaced Persons in Thailand passive surveillance data for Maela showed that the median monthly incidence of URTI was 51.8/1,000 persons (range 30.8–66.1 persons) during May–October 2009; incidence peaked in August. For LRTI, median monthly incidence was 32.4/1,000 persons (range 20.0–37.3), and incidence peaked in September. For the same months of 2008, median monthly incidence was 36.1/1,000 persons (range 18.4–50.8 persons) for URTI and 22.4/1,000 persons (range 11.2–49.9 persons) for LRTI (Figure 4) (R. Sedhain, pers. comm).

## Discussion

Our study demonstrates that influenza virus infections are common etiologic agents of respiratory infection in a Southeast Asian refugee population living in crowded conditions. During the 6 months of surveillance in 2009, influenza A viruses were detected by rRT-PCR in 23% of clinical pneumonia and 20% of ILI cases sampled, representing a considerable impact that this vaccine-preventable disease has among patients with ARI.

Maela is an overcrowded and relatively closed refugee camp and therefore might be considered an ideal location for a novel influenza virus to cause an explosive outbreak. However, the number of confirmed cases indicated that no major outbreak occurred in 2009. After the first case of pandemic (H1N1) 2009 was identified in August, these cases increased modestly in September, then substantially declined during October. Overall, only 25% of all influenza A viruses were determined to be the pandemic strain. However, supportive data show a change of the predominant influenza virus. In late August 2009, seasonal influenza A (H1N1) was the predominant circulating virus; during the subsequent 2 months, only cases of pandemic (H1N1) 2009 were detected. During May–August, the incidence of LRTI and URTI in cases captured by the passive surveillance system was higher each month in 2009 than in 2008. The rates of URTI were similar in September and October of both years, whereas the LRTI rate was higher in October 2008 than in October 2009. Pandemic (H1N1) 2009 did not clearly increase in case-patients with ARI after its first detection in the camp in August 2009. However, surveillance did not capture mild infections that did not result in visits to the outpatient department.

The occurrence of most influenza A infections in patients who had pneumonia most likely reflects a sampling bias, although influenza is a generally underrecognized cause of pneumonia in the tropics (*24*). ILI is not a routinely used diagnosis for the clinic staff at Maela, so most of the ILI case-patients likely were not interviewed and sampled. However, when influenza A was identified, pandemic (H1N1) 2009 case-patients were less likely than seasonal influenza case-patients to have been hospitalized. This information suggests that, in this population, illness caused by pandemic (H1N1) 2009 was no more severe than illness associated with seasonal influenza A. Several confounding factors, unrelated to the innate pathogenicity of the viruses, may account for this finding: 1) the timing of the modification of case definitions in relation to the appearance of pandemic (H1N1) 2009; 2) differences in age distribution; and 3) presence of underlying illnesses in the patient groups. Data regarding underlying medical conditions were not collected as part of this surveillance so the effect of other conditions cannot be assessed. To prevent spread of infection, public health systems may request persons with ILI to self-quarantine, which might result in underestimation of the number of cases identified in clinic- or hospital-based surveillance systems. During the 2009 influenza season, announcements regarding influenza and the need for good hygiene were made on the Maela public address system; healthcare workers reinforced these messages by home visits. Whether this intervention had any effect on health-seeking behavior remains unclear. An influenza triage system was in operation at the hospital, but our surveillance staff had access to patients seen and treated in this area.

Our study has several limitations. Most importantly, not every patient eligible for sampling was included, frequently because the patient refused or clinic staff failed to identify patients with illnesses that met the case criteria. These data were not recorded, so the effect of this bias cannot be estimated. As previously discussed, ILI is not a frequently used diagnosis outside this surveillance program, and most cases with this clinical syndrome were diagnosed as common cold. Many of the ILI cases documented were miscategorized in the clinic as pneumonia but were subsequently found not to meet the case definition, explaining the presence of persons hospitalized with ILI. Overall, these factors may bias toward sampling of case-patients who had more severe symptoms. Also, screening took place in only 1 of the 2 hospital outpatient clinics. However, because both are general clinics, the impact of this screening is likely to be reflected in the absolute number of cases detected rather than in the proportion of ILI and pneumonia cases caused by influenza viruses. Regarding laboratory data, the likelihood of confirmation of influenza infection is associated with the clinical case definitions in use: the strict ILI case definition used in our surveillance has a sensitivity of 98.4%–100% but a specificity of only 7.1%–12.9% (*25*). In another study, the probability of having a positive influenza virus PCR was directly related to magnitude of fever (*26*). Therefore, given the bias toward severe cases, we may have considerably underestimated the impact of influenza in Maela.

As a result of the limitations noted above, we could not directly calculate the incidence of influenza infections in the Maela population. Also, given the mobile nature of refugee populations, calculating accurate incidence rates is difficult, although the monthly census in Maela enabled generation of relatively accurate figures for this population. Therefore, because we detected an influenza virus in 23% of case-patients who had pneumonia during May–October 2009, we believe the virus may have been responsible for 7 pneumonia episodes per 1,000 population per month (32.4 cases × 23%), which equates to ≈900 influenza-associated pneumonia cases during the 3-month influenza season, largely because of seasonal influenza. For comparison, in 2 rural Thai provinces during 2008, influenza virus infection was associated with 18.4% of hospitalized case-patients who had clinical pneumonia (minimum incidence of 134.4/100,000 population) (*27*). Given the likely health inequalities between our refugee population and rural provinces in Thailand, direct comparison of these datasets is difficult. However, the incidence of influenza-associated pneumonia in Maela was ≈5× higher than in the Thai provinces (*27*).

Population structure, such as the number of young children and elderly persons, may account for some of this difference, because the incidence of influenza infection is highest in these age groups. As with ILI, the case definitions used may have affected the data or the use of different laboratory confirmation tests for influenza infection may have resulted in considerable variation in disease rates between studies; the study in Thailand used RT-PCR for laboratory confirmation. Although the rates of influenza-associated pneumonia were different in the refugee camp, the proportions of pneumonia cases associated with influenza were similar (23% vs. 18%).

Methods of preventing or mitigating influenza outbreaks in a community include vaccination; use of antiviral drugs; and basic infection control measures, particularly good respiratory etiquette, hand washing, and social distancing (*28*). The World Health Organization has devised a specific influenza pandemic preparedness and mitigation plan for refugee and displaced populations, but implementation requires the coordinated efforts of healthcare providers (frequently nongovernmental organizations) and governments to ensure that control measures are available and used effectively (*29*). Because resources are likely to be strained during an influenza pandemic, refugee and displaced populations might not be adequately represented in a country’s pandemic preparedness plan. Availability of items required to control influenza transmission (personal protective equipment, vaccines, and antiviral medication) may be limited for this population without robust planning at the local and national levels. In addition to pandemic preparedness, camp administrators and donor agencies should consider routine vaccination for seasonal influenza in these populations.

Continuation and refinement of this surveillance as the pandemic continues may provide further insight into the epidemiology of influenza in resource-poor rural Asian populations. Work such as this solidifies the need of inclusion of refugee populations in influenza vaccine strategies and pandemic planning.

## References

[1] Dawood FS, Jain S, Finelli L, Shaw MW, Lindstrom S, Garten RJ, Emergence of a novel swine-origin influenza A (H1N1) virus in humans. N Engl J Med. 2009;360:2605–15. 10.1056/NEJMoa0903810 19423869

[2] World Health Organization. World now at the start of 2009 influenza pandemic 2009 [cited 2009 Oct 22]. http://www.who.int/mediacentre/news/statements/2009/h1n1_pandemic_phase6_20090611/en/index.html

[3] World Health Organization. Pandemic (H1N1) 2009—update 72. 2009 [cited 2009 Nov 1]. http://www.who.int/csr/don/2009_10_30/en/index.html

[4] Ministry of Public Health (MOPH) Thailand. Influenza A. (H1N1). 2009 [cited 2009 Oct 22]. http://203.157.15.4/Flu/situation/y52/flu_200910141359.pdf

[5] World Health Organization. South East Asia Regional Office (SEARO). Pandemic H1N1 2009—Myanmar [cited 2010 Apr 15]. http://www.searo.who.int/EN/Section10/Section2562_15102.htm

[6] Kumar A, Zarychanski R, Pinto R, Cook DJ, Marshall J, Lacroix J, Critically ill patients with 2009 influenza A (H1N1) infection in Canada. JAMA. 2009;302:1872–9. 10.1001/jama.2009.1496 19822627

[7] Viboud C, Alonso WJ, Simonsen L. Influenza in tropical regions. PLoS Med. 2006;3:e89. 10.1371/journal.pmed.0030089 16509764 PMC1391975

[8] Turner P, Watthanaworawit W, Carrara V, Nosten F. One year of acute respiratory infection surveillance in migrant and refugee populations on the Thai–Burmese border. XI International Symposium on Respiratory Viral Infections. Bangkok, Thailand; 2009 Feb 19–22.

[9] Ahmed J, Bunei M, Kahi V, Pruess F, Njenga K, Muthoka P, Establishing influenza surveillance in two refugee camps in Kenya, 2006–2008. XI International Symposium on Respiratory Viral Infections. Bangkok, Thailand; 2009 Feb 19–22.

[10] Simmerman JM, Uyeki TM. The burden of influenza in East and South-East Asia: a review of the English language literature. Influenza Other Respir Viruses. 2008;2:81–92. 10.1111/j.1750-2659.2008.00045.x 19453467 PMC4634698

[11] Simmerman JM, Thawatsupha P, Kingnate D, Fukuda K, Chaising A, Dowell SF. Influenza in Thailand: a case study for middle income countries. Vaccine. 2004;23:182–7. 10.1016/j.vaccine.2004.05.025 15531035

[12] Dapat C, Saito R, Kyaw Y, Naito M, Hasegawa G, Suzuki Y, Epidemiology of human influenza A and B viruses in Myanmar from 2005 to 2007. Intervirology. 2009;52:310–20. 10.1159/000237738 19776616

[13] Hasegawa G, Kyaw Y, Danjuan L, Saito R, Suzuki H, Cho TM, Influenza virus infections in Yangon, Myanmar. J Clin Virol. 2006;37:233–4. 10.1016/j.jcv.2006.08.003 16971178

[14] Hasegawa G, Kyaw Y, New HM, Danjuan L, Saito R, Suzuki H, Epidemiological study of influenza virus infections in Yangon, Myanmar. Trop Med Health. 2006;34:3–6. 10.2149/tmh.34.3 16971178

[15] Office of the United Nations High Commissioner for Refugees (UNHCR). 2008 global trends: refugees, asylum-seekers, returnees, internally displaced and stateless persons. 2009 [cited 2009 Oct 22]. http://www.unhcr.org/4c11f0be9.html

[16] Ezard N, Gupta RK. Influenza pandemic plans: what about displaced populations? Lancet Infect Dis. 2006;6:256–7. 10.1016/S1473-3099(06)70444-3 16631541

[17] Truman BI, Tinker T, Vaughan E, Kapella BK, Brenden M, Woznica CV, Pandemic influenza preparedness and response among immigrants and refugees. Am J Public Health. 2009;99(Suppl 2):S278–86. 10.2105/AJPH.2008.154054 19461109 PMC4504387

[18] Committee for Coordination of Services for Displaced Persons in Thailand (CCSDPT). Burmese border refugee sites with population figures: February 2008. 2009 [cited 2009 Oct 22]. http://www.ccsdpt.org/download/border_map&populations.pdf

[19] World Health Organization. Integrated management of childhood illness handbook. 1st ed. Geneva: The Organization; 2005.

[20] Centers for Disease Control and Prevention. CDC protocol of realtime RT-PCR for influenza A (H1N1) 2009 [cited 2009 Oct 22]. http://www.who.int/csr/resources/publications/swineflu/realtimeptpcr/en/index.html

[21] Maertzdorf J, Wang CK, Brown JB, Quinto JD, Chu M, de Graaf M, Real-time reverse transcriptase PCR assay for detection of human metapneumoviruses from all known genetic lineages. J Clin Microbiol. 2004;42:981–6. 10.1128/JCM.42.3.981-986.2004 15004041 PMC356857

[22] Emery SL, Erdman DD, Bowen MD, Newton BR, Winchell JM, Meyer RF, Real-time reverse transcription–polymerase chain reaction assay for SARS-associated coronavirus. Emerg Infect Dis. 2004;10:311–6. 15030703 10.3201/eid1002.030759 PMC3322901

[23] Pan American Health Organization. Epidemiological calendar 2000. Epidemiol Bull. 1999;20:13.10829837

[24] Brooks WA, Goswami D, Rahman M, Nahar K, Fry AM, Balish A, Influenza is a major contributor to childhood pneumonia in a tropical developing country. Pediatr Infect Dis J. 2010;29:216–21. 10.1097/INF.0b013e3181bc23fd 20190613

[25] Thursky K, Cordova SP, Smith D, Kelly H. Working towards a simple case definition for influenza surveillance. J Clin Virol. 2003;27:170–9. 10.1016/S1386-6532(02)00172-5 12829039

[26] Boivin G, Hardy I, Tellier G, Maziade J. Predicting influenza infections during epidemics with use of a clinical case definition. Clin Infect Dis. 2000;31:1166–9. 10.1086/317425 11073747

[27] Simmerman JM, Chittaganpitch M, Levy J, Chantra S, Maloney S, Uyeki T, Incidence, seasonality and mortality associated with influenza pneumonia in Thailand: 2005–2008. PLoS ONE. 2009;4:e7776. 10.1371/journal.pone.0007776 19936224 PMC2777392

[28] Monto AS. The risk of seasonal and pandemic influenza: prospects for control. Clin Infect Dis. 2009;48(Suppl 1):S20–5. 10.1086/591853 19067611

[29] World Health Organization. Pandemic influenza preparedness and mitigation in refugee and displaced populations. WHO guidelines for humanitarian agencies 2009 [cited 2009 Oct 23]. http://www.who.int/csr/resources/publications/swineflu/pandemic_preparedness_refugee/en/index.html

